# Antibody response to symptomatic infection with SARS-CoV-2 Omicron variant viruses, December 2021—June 2022

**DOI:** 10.1101/2023.11.17.23298700

**Published:** 2023-11-18

**Authors:** Ryan Sandford, Ruchi Yadav, Emma K. Noble, Kelsey Sumner, Devyani Joshi, Sara Y. Tartof, Karen J. Wernli, Emily T. Martin, Manjusha Gaglani, Richard K. Zimmerman, H. Keipp Talbot, Carlos G. Grijalva, Edward A. Belongia, Christina Carlson, Melissa Coughlin, Brendan Flannery, Brad Pearce, Eric Rogier

**Affiliations:** 1 Centers for Disease Control and Prevention, Atlanta, GA, USA; 2 Oak Ridge Institute for Science and Education, Oak Ridge, TN, USA; 3 Rollins School of Public Health, Atlanta, GA, USA; 4 Kaiser Permanente Southern California, Department of Research & Evaluation; 5 Department of Health Systems Science, Kaiser Permanente Bernard J. Tyson School of Medicine, Pasadena, CA, USA; 6 Kaiser Permanente Washington Health Research Institute, Seattle, WA, USA; 7 University of Michigan School of Public Health, Ann Arbor, MI, USA; 8 Baylor Scott & White Health, Temple, TX, USA; 9 Texas A&M University College of Medicine, Temple, TX, USA; 10 University of Pittsburgh, Pittsburgh, PA, USA; 11 Vanderbilt University Medical Center, Nashville, TN, USA; 12 Marshfield Clinic Research Institute, Marshfield, WI, USA

**Keywords:** COVID-19, SARS-CoV-2 infection, omicron subvariants, immune response

## Abstract

To describe humoral immune responses to symptomatic SARS-CoV-2 infection, we assessed immunoglobulin G binding antibody levels using a commercial multiplex bead assay against SARS-CoV-2 ancestral spike protein receptor binding domain (RBD) and nucleocapsid protein (N). We measured binding antibody units per mL (BAU/mL) during acute illness within 5 days of illness onset and during convalescence in 105 ambulatory patients with laboratory-confirmed SARS-CoV-2 infection with Omicron variant viruses. Comparing acute- to convalescent phase antibody concentrations, geometric mean anti-N antibody concentrations increased 47-fold from 5.5 to 259 BAU/mL. Anti-RBD antibody concentrations increased 2.5-fold from 1258 to 3189 BAU/mL.

## INTRODUCTION

Humoral immune responses to infection with SARS-CoV-2 include production of immunoglobulin G (IgG) antibodies that bind to spike (S) glycoprotein, including the receptor binding domain (RBD) within S, as well as the nucleocapsid (N) protein. Virus neutralization titers and antibodies that bind to RBD and other S protein epitopes have been associated with protection against symptomatic infection with ancestral and pre-Omicron SARS-CoV-2 variants [1–4]. Because antibodies to N protein are not elicited by U.S.-licensed COVID-19 vaccines, the presence of anti-N antibodies can be an indicator of past SARS-CoV-2 infection among vaccinated and unvaccinated individuals [5, 6]. Elevated levels of anti-N antibody may indicate more recent SARS-CoV-2 infection [7]. As new SARS-CoV-2 variants have emerged, serologic assays that quantify IgG antibody binding to spike and nucleocapsid protein antigen have been used to evaluate humoral response to SARS-CoV-2 infection [8]. Post-infection antibodies may reduce risk of re-infection with new SARS-CoV-2 variants, suppress viral replication and reduce COVID-19 disease severity following re-infection [2, 8].

We previously showed that anti-N antibody seropositivity modified COVID-19 mRNA vaccine effectiveness against symptomatic SARS-CoV-2 infection with SARS-CoV-2 Delta and Omicron variants [9]. Among acutely ill patients, higher levels of binding antibodies against ancestral spike protein reduced the odds of testing positive for SARS-CoV-2 Delta and Omicron variants. Here, we assessed humoral immune response to SARS-CoV-2 infection comparing acute- and convalescent-phase IgG antibody levels against N and ancestral spike RBD antigens among case patients infected during Omicron-predominant variant periods from December 2021 through June 2022.

## METHODS

### Study population and sample collection

Between December 2021 and June 2022, respiratory swabs and acute-phase dried blood spots on filter paper were obtained <5 days after symptom onset from ambulatory patients with respiratory illness enrolled in the US Influenza Vaccine Effectiveness Network, as previously described [9, 10]. Patients who tested positive for SARS-CoV-2 by nucleic acid amplification in respiratory specimens were scheduled for convalescent-phase blood sample collection at 21–56 days after enrollment. Data collected from enrolled participants included patient age, date of illness onset, symptoms associated with COVID-like illness, self-reported presence of specified underlying medical conditions, documented COVID-19 vaccination history including dates of COVID-19 vaccination, self-reported laboratory-confirmed COVID-19 <90 or ≥90 days before current illness or electronic medical record of a positive COVID-19 test.

### Serologic assays

Methods for estimating SARS-CoV-2 binding antibody concentration from blood spots have been previously published [11, 12]. Dried blood spot specimens were tested for immunoglobulin G (IgG) antibodies against SARS-CoV-2 recombinant antigens representing ancestral spike protein RBD and nucleocapsid protein using a validated multiplex bead assay (FlexImmArray^™^ SARS-CoV-2 Human IgG Antibody Test, Tetracore, Rockville, MD) on a Luminex MAGPIX instrument with LX200 flow analyzer (Luminex Corporation, Austin, TX). Eluted specimens were diluted 1:300 and individual specimen median fluorescence intensity (MFI) ratios were calculated compared to antigen-specific human IgG calibrator serum. MFI units were standardized to binding antibody units per mL (BAU/mL) against World Health Organization (WHO) international standards [10, 13].

### Statistical analysis

Antibody concentrations in BAU/mL were log-transformed (log 10) for estimation of geometric mean concentrations (GMC) and 95% confidence intervals (CI) and back-transformed for plots by BAU/mL concentrations. Geometric mean fold rise in bAb concentration was estimated as the geometric mean ratio of bAb concentrations measured at enrollment and follow-up. Fold rise in anti-N and anti-RBD antibody concentrations represented response to acute SARS-CoV-2 infection. Associations between antibody fold rise and patient characteristics, vaccination status, baseline N antibody serostatus, and prior positive COVID-19 test were assessed by t-test or ANOVA. Statistical analysis was performed using R version 4.0.3 (R Foundation for Statistical Computing, Vienna, Austria).

## RESULTS

A total of 105 SARS-CoV-2 positive participants had acute-phase blood specimens collected a median of 2 days (range: 0–5) and convalescent-phase specimens collected a median of 36 days (range: 23–61) after symptom onset. Of 48 SARS-CoV-2 positive specimens with genomic sequencing, 43 (90%) belonged to Omicron BA.1 (n= 5) or BA.2 (n=38) lineages, with five sequences belonging to BA.2.12.1 (n=3), BA.4 (n=1) and BA.5 (n=1) lineages. Among 105 case patients with paired specimens, 36% were aged <40 years, 17% were >65 years, 57% were female and 23% had an underlying health condition (Table 1).

Among 7 (7%) patients with ≥1 electronic health record documented prior COVID-19 positive laboratory PCR test, median time since most recent positive COVID-19 test was 351 days (range: 64–758). Among 101 patients who had received at least two doses of COVID-19 mRNA vaccine, median time since receipt of most recent mRNA vaccine dose was 175 days (range: 33–382); 11 had received a booster dose <90 days prior to their enrollment in the study (measured between the date of booster and date of illness onset).

Anti-N and anti-RBD geometric mean fold increases were not associated with days from symptom onset to specimen collection (data not shown). For both antigens, geometric mean fold rise from acute- to convalescent-phase bAb concentrations were similar by patient age, sex, or presence of underlying medical conditions.

In acute-phase specimens, anti-N bAb concentrations were low (GMC: 5.5 BAU/mL, 95% CI: 4.3–7.1; Figure); 72 (69%) participants had anti-N bAb concentrations below the seropositivity threshold of 6.9 BAU/mL. From enrollment to follow-up, anti-N bAb concentration increased 47-fold (CI: 34–66), with convalescent-phase GMC of 259 BAU/mL (CI: 201–335). Among participants with anti-N bAb levels below 6.9 BAU/mL at enrollment, concentrations increased 80-fold (CI: 59–109), versus 15-fold (CI: 7–30) among participants with acute-phase concentrations ≥ 6.9 BAU/mL (*p*-value=0.007). Among four unvaccinated case patients, anti-N bAb concentrations increased 116-fold (95% CI: 7–1998). Patients with prior documented positive COVID-19 tests trended towards having higher convalescent-phase anti-N bAb concentrations than patients without prior positive COVID-19 tests (GMC: 983 [95% CI:546–1771] vs 236 [95% CI: 181–307].

Geometric mean anti-spike RBD bAb concentrations were 1258 (CI: 924–1712) and 3189 (CI: 2639–3853) in acute- and convalescent phase specimens, respectively; mean fold RBD bAb rise was 2.5 (CI: 1.9–3.3; Table; Figure). Geometric mean fold rise in RBD bAb was higher among patients who had received 2 mRNA vaccine doses (7.9 [CI: 2.6—24.3]) versus ≥3 doses (2.0 [CI: 1.6—2.5]). Acute- and convalescent-phase anti-RBD bAb levels were low among four unvaccinated case patients (GMC: 16.0 [CI: 0.2–1557] acute and 149.0 [CI: 0.3—273.1] convalescent).

## DISCUSSION

Among symptomatic patients infected with SARS-CoV-2 Omicron variant viruses, most of whom had been previously vaccinated, we observed marked differences in antibody levels against nucleocapsid protein and ancestral spike RBD antigen measured during acute illness and convalescence. The 47-fold increase in IgG antibodies against SARS-CoV-2 nucleocapsid protein indicated a strong humoral response to infection with Omicron variant viruses, mainly BA.1 and BA.2. Baseline seronegative patients had a higher mean fold rise in anti-nucleocapsid antibodies than seropositive patients. Increased convalescent anti-N bAb levels among baseline seropositive patients suggests boosting of immune responses acquired from past infection; this boosting was also observed among patients with ≥1 documented prior COVID-19 positive test. In contrast, we observed modest 2.5-fold increase in antibodies against ancestral spike RBD antigens. Among vaccinated cases, lower baseline anti-RBD antibody concentrations among patients who had received 2 versus ≥3 mRNA vaccine doses were associated with greater antibody response but similar convalescent antibody concentrations. While anti-RBD bAb levels correlate with protection from infection with pre-Omicron variants [1, 3, 4], antibody levels measured against Omicron variants are greatly reduced [8, 14].

Dried blood spots for SARS-CoV-2 serology were collected as part of a COVID-19 vaccine effectiveness study using a test-negative design. Because antibody levels measured during acute illness reflect an estimation of antibody levels close to the time of infection rather than response to current infection, comparison between antibody levels among case patients and SARS-CoV-2 uninfected patients may provide a measure of correlates of risk [15]. However, data, though limited, suggests that vaccination status and pre-existing neutralizing antibodies may affect anti-N antibody response by altering viral load in early illness [8, 16]. Measurement of immune response to infection and antibody levels after convalescence could improve understanding of vaccinated cases and hybrid immunity [17].

The study had several limitations. The serologic assay used in our study contained ancestral SARS-CoV-2 antigens and serum was quantified using the WHO international serum standards from early in the COVID-19 response. Against pre-Omicron variants, virus neutralization titers and IgG antibody concentrations were associated with protection; however, antibody binding and neutralization activity was lower against Omicron variants [2, 8, 18]. In addition, anti-N bAb seropositivity cut-off values were based on mean fluorescence intensity using serum standards rather than blood spots [10, 13]. All case patients included in this study had mild illness; baseline antibody levels and immune responses may differ among patients with severe or prolonged SARS-CoV-2 infection [19]. Among patients with past infection, initial anti-N bAb concentrations may have waned below the seropositivity thresholds [20, 21]. Finally, this analysis included only four unvaccinated case patients, limiting ability to compare unvaccinated infections and reinfections with vaccine breakthrough infections.

Serologic assays that quantify anti-SARS-CoV-2 specific antibody levels during and following acute infection may provide information on incidence and recency of infection. As new SARS-CoV-2 variants emerge, frequent updates to serologic antigens will be needed to quantify binding IgG antibody levels that correlate with immune protection [14]. Observational studies will be critical for evaluating immune responses to COVID-19 vaccination and infection.

## Figures and Tables

**Figure:**
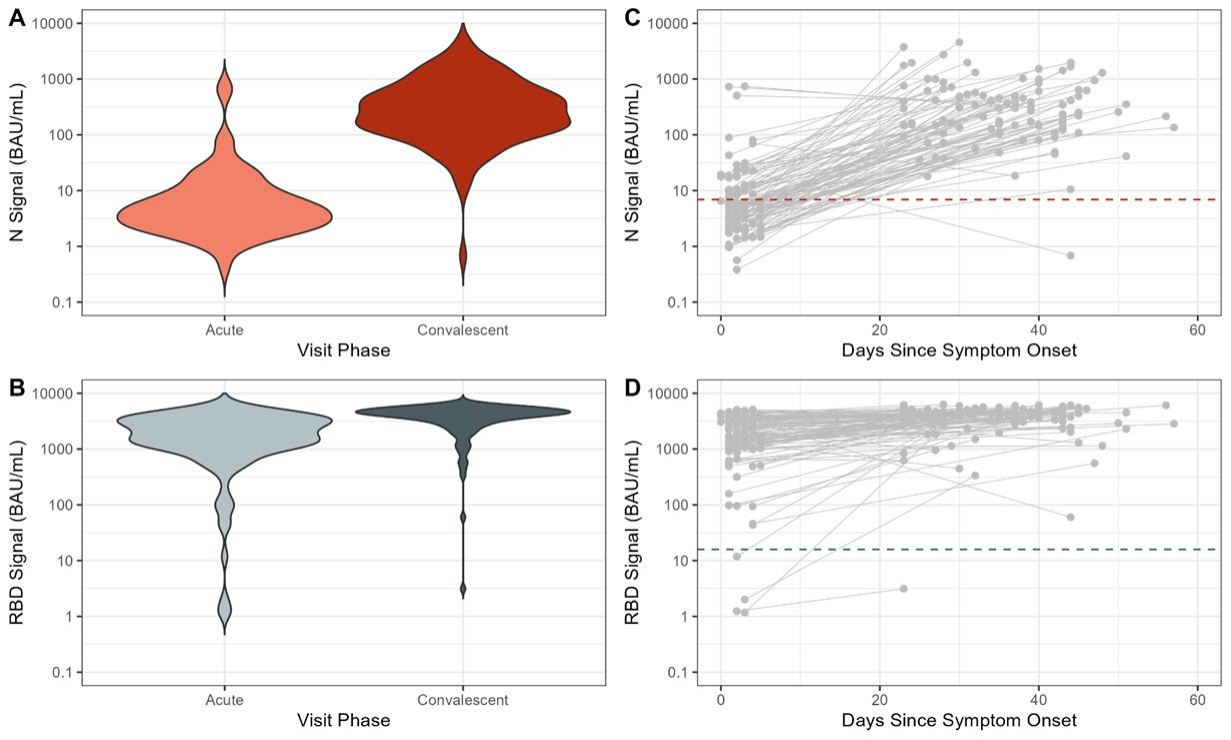
Concentrations of antibodies against SARS-CoV-2 nucleocapsid (N) and ancestral spike protein receptor binding domain (RBD) antigens during acute and convalescent phases of symptomatic COVID-19 associated with Omicron variant virus infection, December 2021—June 2022. (**A**) Antibody concentrations (in binding antibody units [BAU]/mL) against SARS-CoV-2 nucleocapsid protein (N) and (**B)** ancestral spike protein receptor binding domain (RBD) observed in acute and convalescent-phase dried blood spot specimens collected from individuals with symptomatic COVID-19; (**C**) Changes in N concentration and (**D**) RBD concentration in days since symptom onset between acute and convalescent specimens from individuals with symptomatic COVID-19; acute and convalescent-phase specimens from the same individual (N=105) are connected by solid gray line.

**Table:** Concentrations and fold increase in binding antibodies against SARS-CoV-2 nucleocapsid and ancestral spike receptor binding domain antigens during acute- and convalescent-phase of symptomatic infection during Omicron-predominant variant period

	Total N = 105 (%)	α-SARS-CoV-2 Nucleocapsid Antibody				α-SARS-CoV-2 Spike Receptor Binding Domain Antibody			

		Acute	Convalescent	Geometric Mean Fold Rise (95% CI)	p-value^1^	Acute	Convalescent	Geometric Mean Fold Rise (95% CI)	p-value^1^
	
		Geometric Mean Concentration, BAU/mL (95% CI)	Geometric Mean Concentration, BAU/mL (95% CI)						

**Overall**	105	5.5 (4.3, 7.1)	259.4 (200.6, 335.4)	47.0 (33.5, 65.9)		1257.8 (923.9, 1712.3)	3188.5 (2638.7, 3853.0)	2.5 (1.9, 3.3)	
**Age Group**					0.815				0.867
0–39 years	38 (36)	5.0 (3.4, 7.4)	256.9 (160.2, 412.0)	51.0 (28.0, 93.0)		1131.0 (618.4, 2068.7)	2837.9 (2091.0, 3851.5)	2.5 (1.4, 4.6)	
40–65 years	49 (47)	6.7 (4.3, 10.5)	279.7 (193.9, 403.3)	41.8 (24.7, 70.9)		1374.4 (965.9, 1955.6)	3706.1 (3147.8, 4363.4)	2.7 (1.9, 3.8)	
> 65 years	18 (17)	4.0 (2.9, 5.6)	215.7 (113.7, 409.3)	54.1 (27.1, 107.8)		1236.4 (463.3, 3299.5)	2707.5 (1154.6, 6349.4)	2.2 (1.3, 3.6)	
**Sex**					0.580				0.350
Female	60 (57)	5.8 (3.9, 8.5)	294.8 (224.8, 386.6)	51.1 (32.8, 79.5)		1080.7 (661.6, 1765.2)	3058.5 (2304.3, 4059.4)	2.8 (1.9, 4.1)	
Male	45 (43)	5.2 (3.9, 7.0)	218.7 (134.2, 356.4)	42.0 (24.4, 72.5)		1539.8 (1124.3, 2108.8)	3370.6 (2648.0, 4290.3)	2.2 (1.5, 3.3)	
**Underlying medical condition** ^2^					0.914				0.770
Yes	24 (23)	4.8 (3.0, 7.7)	222.7 (130.2, 380.8)	46.6 (24.6, 88.0)		1505.0 (995.7, 2274.6)	3705.4 (3042.7, 4512.3)	2.5 (1.6, 3.8)	
No	76 (72)	5.6 (4.1, 7.8)	273.7 (199.5, 375.6)	48.5 (31.6, 74.4)		1112.0 (742.0, 1666.3)	2970.4 (2303.4, 3830.4)	2.7 (1.9, 3.8)	
**COVID-19 vaccination status**					0.525				**<0.001**
Unvaccinated	4 (4)	9.8 (0.8, 125.0)	1133.6 (631.7, 2034.2)	115.5 (6.7, 1998.0)		16.0 (0.2, 1556.6)	149.0 (2.4, 9353.8)	9.3 (0.3, 273.1)	
2 mRNA doses	14 (13)	3.9 (1.3, 11.9)	214.3 (120.5, 380.9)	55.2 (17.6, 173.2)		414.6 (148.1, 1160.5)	3268.3 (2293.0, 4658.4)	7.9 (2.6, 24.3)	
>3 mRNA doses	87 (83)	5.7 (4.4, 7.3)	250.0 (186.8, 334.5)	43.9 (30.4, 63.4)		1837.7 (1473.7, 2291.6)	3656.2 (3190.6, 4189.7)	2.0 (1.6, 2.5)	
**Baseline α-SARS-CoV-2 nucleocapsid antibody serostatus** ^3^					**<0.001**				**0.001**
Seropositive	33 (31)	24.0 (15.4, 37.4)	350.5 (198.2, 620.0)	14.6 (7.0, 30.3)		2065.3 (1211.5, 3520.9)	2685.7 (1881.4, 3834.1)	1.3 (0.8, 2.1)	
Seronegative	72 (69)	2.8 (2.4, 3.2)	226.0 (171.8, 297.1)	80.3 (59.4, 108.5)		1002.0 (689.3, 1456.8)	3449.4 (2750.9, 4325.3)	3.4 (2.5, 4.7)	
**Prior COVID-19 positive test**					0.754				0.710
Yes	7 (7)	33.1 (7.1, 153.8)	983.4 (545.9, 1771.2)	29.7 (4.5, 196.5)		1476.3 (326.9, 6665.9)	2928.8 (1414.9, 6062.7)	2.0 (0.8, 4.9)	
No	98 (93)	4.9 (3.8, 6.2)	235.8 (181.2, 307.0)	48.5 (34.3, 68.7)		1243.5 (902.3,1713.5)	3207.9 (2628.6, 3914.9)	2.6 (1.9, 3.4)	

Abbreviations: BAU, Binding Antibody Units

1 Student t-test or ANOVA test of difference between log transformed geometric mean fold rise; p<0.05 defined as statistically significant difference between groups.

2 Self reported presence of a serious chronic medical condition such as heart disease, lung disease, diabetes, cancer, liver or kidney disease, immune suppression, or high blood pressure. Missing n=5.

3 Seropositivity (nucleocapsid BAU/mL ≥ 6.9) based on conversion from mean fluorescence intensity ratio ≥1.2 of sample to calibrator serum in multi-bead assay to BAU/mL using WHO standard serum panel.

## References

[1] Benkeser D, Fong Y, Janes HE, Immune correlates analysis of a phase 3 trial of the AZD1222 (ChAdOx1 nCoV-19) vaccine. NPJ Vaccines 2023; 8:36. 36899062 10.1038/s41541-023-00630-0 PMC10005913

[2] Benkeser D, Montefiori DC, McDermott AB, Comparing antibody assays as correlates of protection against COVID-19 in the COVE mRNA-1273 vaccine efficacy trial. Sci Transl Med 2023; 15:eade9078. 37075127 10.1126/scitranslmed.ade9078 PMC10243212

[3] Fong Y, Huang Y, Benkeser D, Immune correlates analysis of the PREVENT-19 COVID-19 vaccine efficacy clinical trial. Nat Commun 2023; 14:331. 36658109 10.1038/s41467-022-35768-3 PMC9851580

[4] Gilbert PB, Montefiori DC, McDermott AB, Immune correlates analysis of the mRNA-1273 COVID-19 vaccine efficacy clinical trial. Science 2022; 375:43–50. 34812653 10.1126/science.abm3425 PMC9017870

[5] Jones JM, Opsomer JD, Stone M, Benoit T, Ferg RA, Stramer SL, Busch MP. Updated US Infection- and Vaccine-Induced SARS-CoV-2 Seroprevalence Estimates Based on Blood Donations, July 2020-December 2021. JAMA 2022; 328:298–301. 35696249 10.1001/jama.2022.9745 PMC9194752

[6] Wiegand RE, Deng Y, Deng X, Estimated SARS-CoV-2 antibody seroprevalence trends and relationship to reported case prevalence from a repeated, cross-sectional study in the 50 states and the District of Columbia, United States-October 25, 2020-February 26, 2022. Lancet Reg Health Am 2023; 18:100403. 36479424 10.1016/j.lana.2022.100403 PMC9716971

[7] Van Elslande J, Oyaert M, Ailliet S, Longitudinal follow-up of IgG anti-nucleocapsid antibodies in SARS-CoV-2 infected patients up to eight months after infection. J Clin Virol 2021; 136:104765. 33636554 10.1016/j.jcv.2021.104765 PMC7891078

[8] Follmann D, Janes HE, Chu E, Kinetics of the Antibody Response to Symptomatic SARS-CoV-2 Infection in Vaccinated and Unvaccinated Individuals in the Blinded Phase of the mRNA-1273 COVID-19 Vaccine Efficacy Trial. Open Forum Infect Dis 2023; 10:ofad069. 36895286 10.1093/ofid/ofad069 PMC9991588

[9] Tartof SY, Xie F, Yadav R, Prior SARS-CoV-2 infection and COVID-19 vaccine effectiveness against outpatient illness during widespread circulation of SARS-CoV-2 Omicron variant, US Flu VE network. Influenza Other Respir Viruses 2023; 17:e13143. 37246146 10.1111/irv.13143 PMC10209645

[10] Sumner K. SARS-CoV-2 Antibody Levels Associated with COVID-19 Protection in Outpatients Tested for SARS-CoV-2, US Flu VE Network, October 2021—June 2022. 2022. 2023. 10.1093/infdis/jiae090 PMC11272097 39052724

[11] Sims MD, Podolsky RH, Childers KL, Dried blood spots are a valid alternative to venipuncture for COVID-19 antibody testing. J Immunol Methods 2023; 513:113420. 36596443 10.1016/j.jim.2022.113420 PMC9804961

[12] Zava TT, Zava DT. Validation of dried blood spot sample modifications to two commercially available COVID-19 IgG antibody immunoassays. Bioanalysis 2021; 13:13–28. 33319585 10.4155/bio-2020-0289 PMC7739400

[13] Mitchell KF, Carlson CM, Nace D, Evaluation of a Multiplex Bead Assay against Single-Target Assays for Detection of IgG Antibodies to SARS-CoV-2. Microbiol Spectr 2022; 10:e0105422. 35647696 10.1128/spectrum.01054-22 PMC9241621

[14] Khoury DS, Docken SS, Subbarao K, Kent SJ, Davenport MP, Cromer D. Predicting the efficacy of variant-modified COVID-19 vaccine boosters. Nat Med 2023. 10.1038/s41591-023-02228-4 36864253

[15] Follmann DA, Dodd L. Immune correlates analysis using vaccinees from test negative designs. Biostatistics 2022; 23:507–21. 32968765 10.1093/biostatistics/kxaa037 PMC9216615

[16] Jager M, Diem G, Sahanic S, Immunity of Heterologously and Homologously Boosted or Convalescent Individuals Against Omicron BA.1, BA.2, and BA.4/5 Variants. J Infect Dis 2023; 228:160–8. 36869832 10.1093/infdis/jiad057 PMC10345468

[17] Duarte LF, Vazquez Y, Diethelm-Varela B, Differential Sars-Cov-2-Specific Humoral Response in Inactivated Virus-Vaccinated, Convalescent, and Breakthrough Subjects. J Infect Dis 2023. 10.1093/infdis/jiad320 PMC10547456 37572355

[18] Lyke KE, Atmar RL, Islas CD, Rapid decline in vaccine-boosted neutralizing antibodies against SARS-CoV-2 Omicron variant. Cell Rep Med 2022; 3:100679. 35798000 10.1016/j.xcrm.2022.100679 PMC9212999

[19] Roltgen K, Powell AE, Wirz OF, Defining the features and duration of antibody responses to SARS-CoV-2 infection associated with disease severity and outcome. Sci Immunol 2020; 5. 10.1126/sciimmunol.abe0240 PMC7857392 33288645

[20] Lea CS, Simeonsson K, Kipp AM, Waning of SARS-CoV-2 Seropositivity among Healthy Young Adults over Seven Months. Vaccines (Basel) 2022; 10. 10.3390/vaccines10091532 PMC9505545 36146610

[21] Van Elslande J, Oyaert M, Lorent N, Lower persistence of anti-nucleocapsid compared to anti-spike antibodies up to one year after SARS-CoV-2 infection. Diagn Microbiol Infect Dis 2022; 103:115659. 35278794 10.1016/j.diagmicrobio.2022.115659 PMC8837483

